# Understanding German Pig Farmers’ Intentions to Design and Construct Pig Housing for the Improvement of Animal Welfare

**DOI:** 10.3390/ani10101760

**Published:** 2020-09-28

**Authors:** Carolin Winkel, Marie von Meyer-Höfer, Heinke Heise

**Affiliations:** Agribusiness Management, Department of Agricultural Economics and Rural Development, University of Göttingen, 37073 Göttingen, Germany; marie.von-meyer@agr.uni-goettingen.de (M.v.M.-H.); heinke.heise@agr.uni-goettingen.de (H.H.)

**Keywords:** farm animal welfare, farmers’ behaviors, housing systems, pig husbandry, Theory of Planned Behavior (TPB), Partial Least Squares (PLS) method

## Abstract

**Simple Summary:**

The husbandry conditions for farm animals are currently being discussed by society and should be improved. In response, some farmers are modernizing their pig housing to offer a higher level of animal welfare than the standard. Moreover, farmers are facing diverse financing and licensing problems when planning new barns. Different studies have already shown that monetary factors and legal requirements often impede farmers from investing in such projects. However, if both of these obstacles for stall construction are excluded, very little is known about the additional factors that influence the behavior of famers seeking to construct pig housing for improving farm animal welfare. Using a model based on the Theory of Planned Behavior, this study investigates the psychological factors underlying farmers’ intentions to construct pig housing based on an online survey among 424 German pig farmers. Utilizing partial least squares path modeling, it is shown for the first time that attitude towards behavior, subjective norm, and direct and indirect experience can explain more than 59.4% of pig farmers’ decisions to construct pig housing with higher farm animal welfare standards. Direct experience and attitude have the strongest influence, while perceived behavioral control does not significantly influence a farmer’s decision. These results, which may also be relevant for other European countries, indicate that in addition to licensing requirements and economic efficiency, the above-mentioned factors also influence the behavior related to barn construction. Farmers, politicians, and stall construction companies should be aware of these results.

**Abstract:**

Improving farm animal welfare requires modifications to the behavior of many stakeholders. Investments in more animal-friendly barns to improve animal welfare have already been made by some farmers. However, more farmers must be persuaded to modernize their barns. The marketing of animal-friendly products is the responsibility of retailers, and consumers have to purchase these products. Currently, little is known about what (and how) underlying psychological factors influence a farmer’s intention to construct pig housing to improve farm animal welfare. Pig farmers in Germany were questioned via an online questionnaire in May 2020 (*n* = 424). Based on the Theory of Planned Behavior (TPB), partial least squares path modeling was used. The constructs: attitude, subjective norm, direct and indirect experience associated with the construction of pig housing substantially influenced the farmers’ behaviors. As expected, the impact of perceived behavioral control on intention was negative but was also very low and only slightly significant. Contrary to expectations, the perceived behavioral control had no significant influence on farmers’ behaviors. Pig farmers who have already rebuilt their pigs’ housing should be motivated to share their experiences to influence their colleagues’ intentions to construct. Our results will encourage policy makers to consider the important role of the different psychological and intrinsic factors influencing pig farmers. Thus, the sustainability of pig farming can be improved by giving politicians a better understanding of farmers’ behaviors.

## 1. Introduction

Twenty-five years ago, society and politics demanded two things above all from pig production in Germany and other European countries: international competitiveness and food safety [1]. The aim was to produce inexpensive and readily available and safe food, as well as to permanently optimize competitiveness [2,3]. However, these expectations of minimum-cost production have changed significantly in recent years. Farmers’ work is increasingly closely monitored and discussed by society, which contributes to the further development and improvement of animal husbandry. Farm animal welfare (FAW) is at the forefront of demands from society (and thus politics). Successful animal husbandry in Germany is increasingly dependent on social acceptance. At the same time, such husbandry must be economical for the pig farmers [2]. To achieve this, farm animal husbandry systems must be adapted to animal welfare, social demands, and economic efficiency. The implementation of higher FAW standards requires complex interventions in the construction of barns. Particularly in pig farming, complex changes in husbandry systems are already underway. Some pig housing have already been adjusted to meet the expectations of higher FAW, and many more will be improved in years to come. Future pig housing is not only about space, but also involves outdoor areas and bedding areas [3,4]. Especially in conventional pig farming, the well-being of pigs can be increased by, for example, bedding, enrichment materials, or outdoor climate stimuli [5,6,7]. Increasingly more pig farmers are rethinking the management of their pigs and FAW [3,8]. In the case of FAW, interventions [9] have to motivate the pig farmers to make changes to their own behavior on behalf of a third party: the pig. This is a different situation from that described in the human health literature. In this way, such interventions encourage people to take steps to improve their own well-being, thereby benefiting themselves directly [9]. Farmers’ attitudes are reflected in their behaviors toward animals, which, in turn, affects animal welfare, behavior, and productivity [10,11]. Kauppinen et al. [12] examined what farmers mean when they talk about improving animal welfare. These definitions included providing animals with a favorable environment, such as a suitable stall. In addition, farmers perceived the instrumental and intrinsic evaluations of animal welfare as attitudinal dimensions [12]. Furthermore, in Jääskeläinen et al. [13], the majority of farmers considered animal welfare to affect productivity and that there are associations between farmer’ attitudes, animal welfare, and productivity. Actions to improve animal welfare also have an economic impact as they enhance pig production [13]. However, learning and opportunity costs arise if pig farmers need to change their housing and management systems, potentially decreasing their monetary benefits and thus reducing their willingness to change the relevant systems [14,15]. However, not only learning and opportunity costs, but also several uncertainties (such as financing, practical uncertainty, a lack of political decisions, legal regulations, and environmental protection in relation to building permits) are unsettling pig farmers.

In 2018, according to the Food and Agriculture Organization (FAO), around 121 million tons of pork were produced worldwide. In terms of export volume, after China and the USA, Germany is the largest exporter in the world, with nearly 3.5 million tons of produced pork per year [16]. To produce competitively at the level of the world market, production has to adapt to the minimum prices in the world market. Low prices are not sufficient for pig farmers to significantly increase FAW in bans, and demands for higher FAW standards at zero cost are not feasible [17,18]. In addition to financial uncertainties, most pig farmers cannot rely on their own expertise, as they have hardly any experience in alternative husbandry methods [19]. Supposed FAW measures that have not been fully researched may also have negative consequences for FAW (e.g., pigs with long tails, group farrowing, and pasture pig production systems) [20,21,22]. In Alarcon et al. [23], other producers, especially those from abroad, seemed to considerably influence farmers’ decision-making. Extensive operational knowledge relevant for the practical implementation of higher FAW standards by conventional farmers repeatedly reveals problems when changing husbandry systems, e.g., free farrowing [24]. Furthermore, for years, an update to the legal regulations on the minimum requirements for future pig husbandry in Germany has been discussed, yet there currently remains little political involvement in such matters. This has led to great legal uncertainty among German pig farmers regarding future pig farming. If the statutory regulations on the minimum requirements for future pig farming were amended, laws directly affecting the construction of pig housing would also have to be adapted [25]. In particular, conflicts of interest with environmental protection can be problematic when constructing pig housing aimed at FAW improvement, especially when approving pig housing [26]. Such obstacles include emissions into the air, soil, or groundwater [26]. Politicians are planning to change the administrative and judicial procedures in Germany to simplify the bureaucracy involved in building barns for pig farmers. In the future, agricultural businesses will benefit from such accelerated administrative and judicial procedures [27,28,29]. Pig farmers expect clear and long-term statements on livestock management and future legal standards from policymakers. In light of all these uncertainties, the construction of pig housing with increased FAW standards is associated with a high degree of uncertainty for pig farmers and is consequently followed by low acceptance.

To date, research has focused on the socio-economic and behavioral aspects of improving animal welfare, but not on the construction of pig housing aimed at specifically improving FAW. The objective pursued by this study, therefore, is to understand the behaviors of farmers related to developing more animal-friendly housing systems. The aim of this study is to identify what (and how) underlying psychological factors influence a pig farmer’s intention to construct pig housing for the improvement of animal welfare, as well as the actual behavior of such farmers, using the conceptual framework of the Theory of Planned Behavior (TPB). In Kauppinen et al. [12], farmers’ intentions to improve animal welfare can best be explained by their attitudes to specific welfare-improving measures using TPB. Germany was selected to conduct the study because it is one of the countries with the highest current FAW developments and focused on future improvements. Although our study is based on a sample of German pig farmers (see Appendix A Table A1), our results are also partially transferable to other countries with similar structures in the pig production sector. However, the legal standards differ between the various countries with high output (pigs). Since these minimum standards are so different, this study would have to be conducted separately for other countries with high outputs. Foreign analyses could, however, be based on the structure of our study. The identification of the relevant determinants will help us understand how more conventional farmers might be persuaded to construct barns with higher FAW standards. The aim of this study is to provide valuable insights into the mechanisms that influence German pig farmers to engage in stall construction. The political promotion of constructing pig housing to improve FAW could be based on this insightful information in the future. This means, for example, that not only could the construction or conversion of pig housing be a factor of influence but also internal attitudes towards the topic of stall construction.

## 2. Conceptual Framework

**Figure 1:**
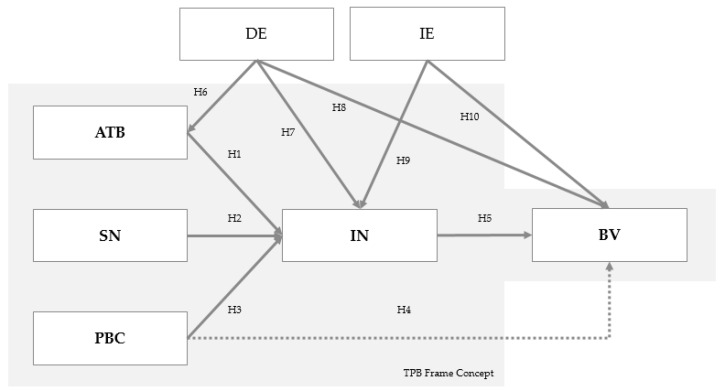
Model used in the partial least squares (PLS) analysis according to Theory of Planned Behavior (TPB). ATB = attitude towards behavior, BV = behavior, DE = direct experience, IE = indirect experience, IN = intention, SN = subjective norm, PBC = perceived behavioral control. Source: Authors’ illustration.

A model based on the TPB (Figure 1) was used to identify the psychological determinants of the decision to construct pig housing to improve FAW. The TPB assumes that the intention to act is the immediate determinant of behavior [30]. People, therefore, act in accordance with their behavioral intentions. These intentions are, in turn, determined by three influencing constructs: “attitude towards behavior”, “subjective norm”, and “perceived behavioral control” [31]. According to this theory, the stronger the intention is to engage in a behavior, the more likely the exercise of that behavior will be [31]. The TPB is popular for its simplicity [32,33,34] and is already often utilized in agri-economic studies analyzing farmers’ decision-making behaviors [35,36,37] and to study FAW [38].

Based on the results of previous studies, further constructs were added to the original TPB model to adapt the model to the research question posed in the present study. In Figure 1, all the constructs and hypotheses used in this study are summarized into one model. The original framework of the TPB, which was first introduced by Ajzen [30], was extended by including direct and indirect experience. From a behaviorist perspective, Eagly and Chaiken [39] consider experience to be an important direct predictor of current behavior in TPB. The two constructs that were added to adjust the TPB model to this study are outside the highlighted TPB framework concept. The respective questions (items) of the constructs can be seen in Appendix A, Table A2. The questions to test the hypotheses were formulated based on validated items from questionnaires previously used for the respective constructs.

“Attitude towards behavior” refers to the degree to which a person has a favorable or unfavorable evaluation in relation to a certain behavior [31]. Individuals form their attitudes based on their perception of a particular subject. This perception can be based on an emotional reaction to the object, supported by values and beliefs, or based on knowledge and/or information [40]. Studies on farmers’ attitudes towards FAW programs [8,12,15,41] can be applied to the conversion or construction of pig housing with higher FAW standards because the conversion or construction of pig housing with higher FAW standards on one’s own farm is necessary to implement higher FAW measures and facilitate participation in animal welfare programs to react to public demands. A positive attitude generally increases the likelihood of performing the behavior [30].

Subjective norm is determined by normative beliefs of the expectations of people who are important to the respondents [31]. For example, do farmers believe that their colleagues or families expect them to construct pig housing to improve FAW and does that belief motivate the farmers to comply with those people [42,43,44]?

The TPB, as an extension of the Theory of Reasoned Action (TRA), includes an additional construct called perceived behavioral control. This construct is also assumed to influence intention and behavior and is equivalent to the difficulty or ease perceived by the individual in performing the behavior. Based on validated items that have already been used in questionnaires, as well as corresponding literature, items for the construct of perceived behavioral control were adapted by the authors to the present topic. The items for perceived behavioral control were largely formulated as obstacles. If obstacles such as a lack of knowledge or too much effort are confirmed [38], their impact is considered to be negative. In TPB, the more favorable these three constructs are, the stronger an individual’s intention to express the analyzed behavior will be [31].

For the construction of pig housing to improve FAW, the intention to construct pig housing to improve FAW is determined firstly by the specific attitude towards pig housing aiming at such improvements, secondly by the perceived expectations of the social environment, and thirdly by expectations of competence or control—i.e., the subjectively perceived ability to construct this pig housing. Based on this, three hypotheses were formulated and tested in this study:

**Hypothesis 1** **(H1).** *the attitude towards the construction of pig housing to improve FAW influences the pig farmer’s intentions to construct pig housing.*

**Hypothesis 2** **(H2).** *the subjective norm influence the farmer’s intention to construct pig housing to improve FAW.*

**Hypothesis 3** **(H3).** *perceived behavioral control influences the farmer’s intention to construct pig housing to improve FAW.*

According to the original TPB framework, perceived behavioral control directly influences the actual adoption of a certain behavior [31]. Intention accounts for the motivation that a person has, while perceived behavioral control refers to the ability of a person to perform that behavior. Even though the intention to perform a certain behavior may be low, higher perceived behavioral control over a certain behavior increases the probability of performing that behavior [31]. According to Ajzen [30], a distinction is made between the perceived and actual control of one’s behavior. Perceived behavioral control captures a person’s beliefs in how easy or difficult it is to engage in a behavior. This relates to the perception of one’s own capabilities and available resources. Actual behavioral control involves external factors. If perceived behavioral control reflects actual behavioral control, it can have a direct effect on behavior. If pig housing with increased FAW standards is considered a new type of housing for most pig farmers, it can be assumed that external technical barriers will negatively influence the implementation of housing with increased FAW standards [38]. This line of argument is applicable to the introduction of pig housing construction to improve FAW. The following hypothesis can thus be formulated:

**Hypothesis 4** **(H4).** *perceived behavioral control influences the farmer’s behavior to construct pig housing to improve FAW.*

In addition, in the basic TPB, another construct directly influences the actual performance of a certain behavior. Intention accounts for the motivation that a person has. A pig farmer who previously had low intention to carry out pig housing construction with increased FAW standards is less likely to carry out this behavior, even if this pig farmer has the ability to complete the construction [31]. Consequently, the following hypothesis is formulated:

**Hypothesis 5** **(H5).** *the intention to construct a pig housing with improved FAW standards influences the farmer’s behavior to construct such a pig housing aiming at improving FAW.*

To adjust the TPB model to this study’s focus, two more constructs were added to the model: direct experience and indirect experience in the design and construction of pig housing with improved FAW standards. For Ajzen and Fishbein [45], experience is a variable external factor. Ajzen and Fishbein [45] assume the influence of experience within the framework of their theory but conceive of experience as being entirely conveyed by convictions and evaluations. To verify this, we examine the direct influence of experience on attitude. Here, we focus only on direct experience. De Lauwere et al. [38] and Garforth et al. [46] noted that experience is a particular factor in decision-making, especially for farmers, and could thus shape farmers’ attitudes.

**Hypothesis 6** **(H6).** *Direct experience with the construction of pig housing to improve FAW influences people’s attitude towards behavior.*

Bentler and Speckart [47] added past behavior (i.e., experience with behavior) as a further predictor for the constructs of TRA. Past behavior will influence present behavior directly and indirectly via intention. Ouellette and Wood [48] gave theoretical reasons for why experience with behavior contributes to the formation of intention. In the studies by Conner and McMillan [49], Drake and McCabe [50], and Banfield and McCabe [51], experience with behavior also contributed to the prediction of intention beyond past the TPB constructs. In our case, the complete execution of the target behavior, i.e., the construction of pig housing with increased FAW standards, cannot be a predictor for the past when the behavior was first considered. Experience with partial aspects of the behavior and the object—in our study pig housing to improve FAW—can be considered a predictor. Possibly, neither the complete execution of the behavior (multiple times) nor experience with the entire behavior is necessary to become behavior-effective. However, experience with single, indirect aspects of the behavior (executing any construction) or its objects (colleagues’ pig housing with increased FAW standards) can certainly allow one to become behavior-effective. For a differentiated investigation, direct experience (one’s own execution of construction for increased animal welfare) is distinguished from indirect experience (reports from a colleague on pig housing construction with increased FAW standards). Direct experience likely has a stronger influence on intention than indirect experience. We assumed that the experiences already gained from the construction of pig housing would exert influence on pig farmers’ behavioral intention and actual adoption. Earlier negative or positive experiences can likewise negatively influence the behavioral intention to construct pig housing and thus actual adoption. The background for these considerations is a survey among farmers changing from individual to group housing for their pregnant sows [38].

**Hypothesis 7** **(H7).** *direct experience with the construction of pig housing for the improvement of animal welfare influences the farmer’s intention to construct pig housing to improve FAW standards.*

**Hypothesis 8** **(H8).** *direct experience with the construction of pig housing to improve FAW standards influences the farmer’s behavior to construct such pig housing.*

**Hypothesis 9** **(H9).** *indirect experience with the construction of pig housing to improve FAW standards influences the farmer’s intention to construct such pig housing.*

**Hypothesis 10** **(H10).** *indirect experience with the construction of pig housing to improve FAW influences the farmer’s behavior to construct such pig housing.*

## 3. Methodology

### 3.1. Sampling and Study Design

For this study, an anonymous online questionnaire was used to analyze 442 conventional and organic pig farmers in Germany who participated in May 2020. Online surveys are considered a cost-efficient, effective, and timesaving method of data collection [52,53,54]. The survey participants were reached via e-mail, as well as through newsletters from a leading German farm management magazine, the Associations of Pig Farmers in different German federal states and the Association of Pig Farmers in Germany. In addition, the survey was distributed by some Chambers of Agriculture and was available on their websites. In Germany, each of the 16 federal states has a chamber of agriculture or a state office for agriculture. We arbitrarily chose pig farmers that were available through our distributors. We characterize our type of sampling as a convenience strategy. The survey questions consisted of open-ended and closed questions. The closed questions had to be answered on five-point Likert scales. The questionnaire consisted of six parts: in Part I, the introductory questions dealt with farm characteristics, such as farm size and pig housing conditions. In Part II, the farmers were asked about new buildings or conversions of their own pig housing. In Part III of the questionnaire, 36 statements were used to determine the farmers’ behaviors towards pig housing construction. They were asked to rate given statements based on five-point Likert scales from “completely agree” to “completely disagree”. The statements had to be answered by both fattening pig farmers and farmers engaged in sow management. The questionnaire determined what farmers understand by “improving animal welfare”. Animal welfare here means measures that go beyond the legal standards. At least the measures of level 1 (of a total of 3) of the state’s animal welfare label for pigs should be fulfilled. Here are the measures again: specifications according to the Tierschutznutztierhaltungs-Verordnung and QS standards; feeding according to legal requirements; farrowing pen with fixation; pregnant sows in group housing from the 28th day after insemination; 20% more space and organic employment material; nesting material in the farrowing pen made of long-fibrous organic materials in the farrowing area; 4-week suckling phase (with at least 25 suckling days); no anesthetic-free castration; permissible alternative procedures: castration with anesthesia, boar fattening, vaccination against boar odor. Upwards, i.e., higher animal welfare measures, there are no limits. Here, animal welfare means measures that go beyond the legal standards. At least the level 1 measures (of a total of 3) of the state’s animal welfare label for pigs in Germany had to be fulfilled. The corresponding measures were added as an information box. The data served as the basis for the structural model and thus as indicators of the constructs analyzed in the TPB model (see Appendix A Table A2). Part IV consisted of questions about a slaughter company in Germany that founded a barn construction cooperative to improve farm animal welfare on a broad basis. In Part V, the participating farmers had to answer socio-demographic questions, such as the economic efficiency of their farm and their age, gender, and education. A prerequisite for answering the research question was that the farmers kept their pigs in an agricultural holding. A filter in the questionnaire prevented the participation of ‘non-pig farmers’. In addition, the data set was adjusted for the participants whose farms are not located in Germany. Furthermore, the data set was checked for missing values, answer patterns, inconsistent answers, and outliers in accordance with the recommendations of Wagner and Hering [54]. After adjusting the data set, the sample contained 424 participants.

### 3.2. Analysis Methods

The Partial Least Squares (PLS) method was used to evaluate the collected data, as this method is particularly suitable to analyze the causal relationships considered in the model presented and has been successfully and repeatedly used in TPB models. During the calculations, which were carried out using the Smart-PLS Version 3.3.0 software, the quality of the measurement model was first tested using various quality criteria for reliability and validity before actual verification of the hypotheses was carried out. The constructs of the adjusted TPB model were latent variables, which were not directly observable. These variables have had to be described through items (empirical indicators) [55]. The aim of the PLS-SEM (structural equation modeling) was to maximize the coefficient of determination (R^2^) of the (target) constructs minimize their unexplained variance [56,57].

### 3.3. Quality Criteria of the Measurement and Structural Model

According to Hair et al. [56], evaluation of the PLS-SEM results was carried out in two consecutive steps: evaluation of the outer model (measurement model) followed by evaluation of the inner model (structural model). The measurement model describes the relationships between the latent variables and the associated items [56]. A distinction was made between the formative and reflective measurement models of the latent variables [58,59]. In the TPB model, it is assumed that all constructs are specified as reflective. Reflective measurement models have an effect relationship where the measurement of items is caused by the constructs [60]. In contrast, the structural model describes the relationships between the constructs themselves [56].

The outer model assessment included evaluation of the reflective constructs to determine their indicator reliability, convergent validity, and discriminant validity [61]. The test criteria included (especially for reflectively specified measurement models) internal consistency reliability (Cronbach’s alpha, composite reliability), convergence validity (indicator reliability, average variance extracted (AVE)), and discriminant validity (Fornell-Larcker criterion, cross loadings, heterotrait-monotrait ratio (HTMT)) [56]. Table 1 gives an overview of the quality criteria for the measurement model.

Indicator reliability was related to the extent of an indicator’s variance, which the construct can explain. Reliability is established if the outer loadings of the indicators are significant and their value exceeds 0.708. To check whether the group of indicators sufficiently describes the construct, Cronbach’s alpha values were determined. By estimating the Cronbach’s alpha and Composite reliability, the internal consistency’s reliability was assessed. These procedures examined whether the indicators assigned to the latent variable measure were the same, i.e., consistent. These values should ideally exceed 0.7 [56]. Composite reliability was used as a measure for internal consistency reliability, for which values between at least 0.7 and 0.95 were acceptable. Convergent validity also refers to the extent to which a latent construct can explain each indicator’s variance and can be assessed through the average variance extracted (AVE). The AVE should be above the level of 0.5, meaning that the variance captured between the constructs and corresponding indicators surpassed the level of variance due to measurement errors [56]. To evaluate discriminant validity, the Fornell–Larcker criterion had to first be met. The Fornell–Larcker criterion is used to assess whether a construct shares greater variance with the items assigned to it than with another construct [56]. Cross-loadings of the indicators were also assessed to achieve discriminant validity. If, for example, the cross-loading on an unassigned construct was too high, the indicator had to be removed. A third way of assessing the validity of discrimination, the HTMT, can be used. The criterion was based on the correlation between the items that measure different latent variables and the correlation between items that measured their own latent variable. Values should not exceed 0.90 [62] to ensure that the constructs are separable from each other.

After the validity and reliability of the measurement model had been assessed sufficiently, the structural model itself was checked. The evaluation of the inner (structural) model served to assess the predictive power of the model regarding the (target) constructs [56]. The test criteria included the significance and magnitude of the path coefficients, the coefficient of determination (R^2^), and the prediction relevance (Stone-Geisser-Q^2^) [56]. The determinate measurement of the R^2^ of the endogenous variable measured the quality of the fit of the regression function to the indicators and determined the proportion of the explained variance [56]. The larger the R^2^ was, the higher the proportion of the explained variance. Since PLS-SEM is a non-parametric estimation method, it was necessary to apply a re-sample bootstrapping method to test the path coefficients’ significance. Bootstrapping is a non-parametric method and was used to create subsamples that were randomly pulled out of the record. In this study, following the recommendations of Hair et al. [56], 5000 bootstrap samples were used to generate t-statistics. To be considered as a meaningful influencing factor, the path coefficients should exceed a value of 0.2. The Stone–Geisser test (Q^2^) was used to determine the predictive validity of the model and was itself determined via the so-called “blind following” procedure [56]. In this procedure, it was systematically assumed that a part of the raw data matrix was missing in the parameter estimation [63]. This procedure clarified how well the collected data could be reconstructed by the model. A Q^2^ > 0 suggested that the model had good predictive validity [56].

## 4. Results and Discussion

### 4.1. Sample Description

In May 2020, 424 pig farmers completed the questionnaire described above (see Appendix A Table A1). In total, 12.2% of the respondents were female. Since about one third of all employees in German agriculture are female, women were somewhat underrepresented in this study [64]. About 75.3% of the respondents were younger than 55 years. The average age of the sample was thus slightly lower than the overall reality in Germany (64.6% younger than 55 years) [64]. Most of the respondents lived in Lower Saxony and North Rhine-Westphalia, where pig farming is most heavily concentrated in Germany [65]. In 92% of the cases, pig farming was the main source of income, and for 8%, it was a secondary source of income. In the national average, 48% of farms are primary holdings, and 52% are secondary holdings [66]. In total, 38.4% of the respondents were foremen, and 47.6% had a university of applied sciences degree or a university degree. According to the results of the 2016 agricultural structure survey, 65% of all agricultural managers had completed at least one vocational training course [64]. At the time of the survey, 49.8% of the respondents were occupied with pig housing conversion or developing a new construction for the improvement of animal welfare. Those who were not involved in pig housing conversion or new constructions (50.2%) noted a lack of political planning security as the main reason for not engaging in FAW development (74.4%). Moreover, 12.5% of these respondents stated that they lacked the financial capacity to change their pig housing.

### 4.2. Evaluation of the PLS Path Model

For the analysis of the extended TPB model, we followed the approach described by Hair et al. [56] and performed the analysis in two steps. First, the measurement model (outer model) was checked, followed by the structural model (inner model). The indicators that were retained in the model over the course of examining the indicator reliability and convergence criteria were attitudes towards behavior (ATB), subjective norms (SN), perceived behavioral control (PBC), direct experience (DE), and indirect experience (ID), with intention (IT) and behavior (BV) for the construction of pig housing (see Appendix A Table A2).

Assessing the constructs for reliability and validity was a prerequisite for the interpretation of the path model [56]. In the present model, the critical threshold of Cronbach’s alpha (0.700) [57,67] was not reached by the constructs of BV (0.662) and PBC (0.618). Nevertheless, in exploratory research projects, values between 0.60 and 0.70 have been considered acceptable [56]. BV was given a presumed value of 0.815 for composite reliability and PBC was given a value of 0.795 for composite reliability. It can be generally assumed that the true value lies generally between Cronbach’s alpha and the composite reliability [56]. Therefore, the internal consistency reliability was taken as given (see Appendix A Table A3).

Convergence validity existed if the indicators assigned to the construct shared a high proportion of variance [56]. Information on this variance was provided by the number of loadings on a construct (indicator reliability). For a construct to have a substantial proportion (50%) of the variance of the indicator, its charge should reach at least 0.700 [63]. In the present model, the lowest loading was found for PCB1 (0.738) (see Appendix A Table A3), which was above the critical threshold. Indicator reliability was thus determined for the model (see Appendix A Table A2). A second quality criterion for assessing convergence validity was the AVE [63]. The quality criterion was also fulfilled, and convergence validity was, therefore, achieved (see Appendix A Table A3).

To assess discriminant validity, the HTMT criterion was focused upon, as recommended by [56]. Except for one value (0.903), all HTMT values of the correlations were below the limit value (see Appendix A Table A4). The ratio of the constructs BV–IN had a value of 0.903, which exceeded the critical threshold and theoretically meant that discriminant validity in the model did not exist according to the HTMT ratio. The two constructs were apparently conceptually very similar. The bootstrapping procedure indicated that the constructs were, however, empirically different. Furthermore, since the Fornell–Larcker criterion and the cross-charge were tested to be positive, the discriminant validity in the model was taken for granted.

**Figure 2:**
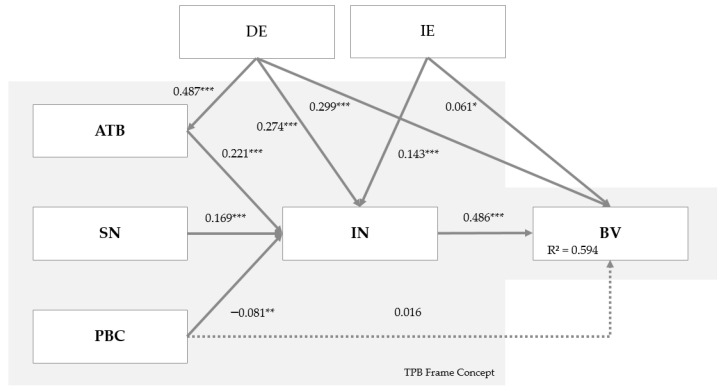
Results of the partial least squares (PLS) analysis: determinants to design and construct pig housing for the improvement of animal welfare. The values are presented as the path coefficients. Significance level: * *p* < 0.1, ** *p* < 0.05, *** *p* < 0.01. Broken line: not significant. ATB = attitude towards behavior, BV = behavior, DE = direct experience, IE = indirect experience, IN = intention, SN = subjective norm, PBC = perceived behavioral control. Source: Authors’ calculations and illustration.

The second step of the quality assessment of the path model was to evaluate of the inner model. Based on this evaluated, we decided whether hypotheses 1–10 (derived in Section 2) could be supported. An overview of the significance of the path coefficients calculated with the PLS algorithm and the hypotheses are given in Appendix A Table A5 and Figure 2. The estimated structural model for the TPB explained 69.4% of the variance for intention and 59.4% of the variance in behavior to construct pig housing for the improvement of animal welfare. A comparison with other studies showed that when applying TPB to agricultural science questions, coefficients of determination of 50% relevant for assessment [38,44,68,69].

The paths ATB (+0.221***), SN (+0.169***), DE (+0.274***), and IE (+0.143***) for pig housing construction had a significantly positive influence on behavioral intention. DE and ATB had the strongest influence, among which ATB had a particularly strong influence. The influence of PBC (–0.081**) was marginal and negative. According to [56], path coefficients below 0.1 are not truly interesting to interpret, even though they are significant. The influence of PBC (+0.016) on BV was again marginal and not significant. Furthermore, DE also had a very strong and significant influence on ATB (+0.487***) and BV (+0.299***). The extent to which IE influences BV (+0.061*) was also tested. However, the influence of IE on BV was small and only slightly significant. Finally, the influence of IN (+0.486***) on BV was determined to be very strong and significant.

H1 described the influence of ATB on the intention of pig farmers to construct pig housing to improve animal welfare. The path coefficient had a significance level of 1%, and the hypothesis was supported. Therefore, the results suggest that the intention of pig farmers to construct pig housing increases if they have a positive attitude towards constructing a pen to improve animal welfare. This result is in line with previous studies showing that attitudes are one of the most influential factors determining the implementation decisions of public relations measures at the individual farm level [70,71,72].

H2 was also be supported. Subjective norm, e.g., perceived social pressure from family and friends, had a positive and statistically significant effect on intention, as expected (Hypothesis 2). Farmers themselves are often unaware of how much the opinions of others influence their own behaviors [38]. According to Nolan et al. [73], individuals tend to deny that other people’s behavior influence their actions. This suggests that people are generally unaware of the influence that social norms have on them. In our study the subjective norm was conceptualized by the overall social pressure perceived from others who are significant—e.g., “People who are important to me (...)” [74]. The pig farmers interviewed perceived that the people relevant to them expected that they should convert their pig housing for the improvement of animal welfare. This result also confirms the results of Ambrosius et al. [75] regarding the course of agricultural decision-making processes. Comparing the path coefficients, the results also indicate that attitudes towards pig housing construction have a higher effect on IN than SN.

Hypothesis H3 relates to the effect that PBC has on IN. Even if the influence is very close to 0, indicating an influence trend, the hypothesis can still be maintained [76]. The path coefficient from PBC to IN deviated from zero with a probability of error that was <5%. In addition, the direct influence of PBC on behavior was very small, as suspected in H4. Furthermore, the path coefficient (PBC → BV = 0.016) did not deviate statistically from zero. Although the direction of the effect was positive, as assumed, H4 could not be supported, which contradicts the TPB model of [30]. The mean values for the perceived behavioral control indicators did not trend towards disagreement, indicating that farmers see no difficulties associated with the construction of pig housing or perceive see difficulties in aspects other than those included in the questions. Evidence for the latter explanation is given by the descriptive evaluation of another question in our questionnaire. We asked about the obstacles that prevented the farmers in question from engaging in a sty conversion or new construction. Those who were not involved in pig housing conversions or new construction (50.2%) rated the lack of political planning security as the main reason (74.4%). Other difficulties or barriers to the conversion and construction of pig housing included problems with permits (43.9%), conflicting objectives with environmental protection (25.6%), a lack of financial capacity (12.5%), and a lack of space for new barns (9.6%). Other reasons were their satisfaction with the current barns (73.4) or the fact that conversions were carried out in the last 10 years (48.3%). These barriers were queried in another part of our questionnaire. Vogelsang [77] also found only a small influence of PBC in a preliminary study. In Vogelsang’s main study, perceived behavioral control was re-assessed due to its inadequate internal consistency. Its contribution to clarifying intention, which was not observed in the preliminary investigation, focused on perceived external obstacles. In our study, the effect of PBC on IN was negative. Since the influence of PBC was only marginal in our study, it can only be cautiously asserted that if a pig farmer classifies perceived behavioral control or barriers to pig housing construction as high, it can be assumed, conversely, that his or her intention to implement pig housing construction will be low. Our results also conflict with some previous applications of TPB in the agricultural sector. For example, de Lauwere et al. [38] found statistically significant effects of PBC on the intention to implement group housing for pigs. Our measurement of PBC, i.e., the indicators applied, differed insignificantly from this previous study. One reason for this difference could be inappropriate operationalization of the construct, which was reflected by the poor internal consistency of the scale (Cronbach’s alpha = 0.618). The fact that the level of PBC did not contribute anything to the behavioral predictions may relate to the blending of two conceptually different aspects of control in this construct. Terry and O’Leary [78] noted that, according to Ajzen and Madden [79], PBC measures the subjective perception of one’s degree of control over the execution of a behavior (a measure of perceived control, e.g., “It is in my power to execute the behavior in question”). An assessment of how easy or difficult it would be to execute the behavior was also measured (self-efficacy expectations, e.g., “I am competent to perform the required behavior”). Therefore, it is not surprising that this construct often cannot be reliably recorded (see Cronbach’s alpha value of 0.618 in our study). A possible solution to this problem could be the decomposition of PBC. Terry and O’Leary [78] attempted this type of decomposition in a study on the prediction of sports exercises. The authors simultaneously surveyed perceived behavioral control as a measure of external control and self-efficacy as a measure of internal control, and different effects of the two constructs on behavioral intention and actual behavior were observed. Terry and O’Leary [78], therefore, concluded that PBC (as a perceived hindrance to the execution of behavior by external factors) and self-efficacy (in terms of internal control factors) should be included as separate variables in the TPB. Further investigations also proved the empirical distinctness of self-efficacy and perceived behavioral control or controllability [80,81,82,83].

H5 stated that a higher IN among pig farmers would have a positive influence on a farmer’s behavior to construct pig housing for the improvement of animal welfare. This hypothesis was maintained, as the path coefficient from IN to BV differs from zero with a probability of error <1%. However, it is important to note the possible lack of discriminant validity between IN and BV (see Appendix A Table A4), which may explain the high value of the path coefficient. However, this relationship between IN and BV was postulated in the original model by Ajzen [30]. This is a central result of this study, as all basic hypotheses from the TPB framework (see Figure 2), limited by H4 (PBC → BV), can be applied to the construction of pig housing for the improvement of animal welfare. Thus, if the intention to construct pig housing is high, this high intention will have a positive effect on actual building behavior. A significant influence of intention on behavior in agricultural decisions was also found in the studies of Ambrosius et al. [75] and Munoz et al. [71].

Hypotheses H6 to H8 examined how DE affects ATB (H6), IN (H7), and BV (H8). The original TPB model was extended by the two constructs of DE and IE. Eagly and Chaiken [39] considered experience as a direct and important predictor of current behavior in the TPB from a behaviorist perspective. Since the corresponding path coefficients all had a positive value at different significance levels, H6 to H8 were supported. Thus, if farmers had direct experience with the construction of pig housing to improve animal welfare, this experience had a positive effect on ATB, IN, and BV, with a probability of error of <1%. The relationship between DE and ATB had the highest path coefficient, with a value of 0.487. Thus, the attitude of pig farmers was significantly determined by direct experience. The total effect of DE on IN corresponded to 0.382 (=0.487 × 0.221 + 0.274), which is greater than the direct effect of DE (0.274) on IN. Similar observations were be made for the effect of DE on BV. The direct effect of DE on BV (0.299) was less than the total effect of DE on BV (0.419). The high total effect indicated the relevance of DE. Our results provide an interesting addition to Ouellette and Wood [48]. In Ouellette and Wood [48], past behavior was also highlighted as experience, and the authors theoretically justified why past behavior would contribute to the formation of intentions.

Hypotheses H9 and H10 relate to the influence of IE on the intention to construct pig housing and actual behavior. According to the model, both hypotheses can be supported since the relationships between IE and IN and BV, respectively, had path coefficients that differed from zero at a significance level of 1% and 10%. However, the path coefficient between IE and BV differed only slightly from zero (although still significantly). According to Dessart et al. [84], contact with experienced farmers who had already adopted certain practices enabled the collection of information that would otherwise be unavailable. There are many uncertainties related to constructing pig housing, as this practice is innovative compared to the status quo of conventional pig farming methods. Contact with pig farmers who have already adopted this practice and have practical experience in applying it can reduce such uncertainty. Seeing other pig farmers implement a practice can motivate more pig farmers to do the same [84]. To facilitate the widespread adoption of pig housing to improve animal welfare, it is, therefore, beneficial to encourage early adopters to communicate the positive aspects of the process. Similar to Vogelsang [77], indirect experience had a smaller contribution than direct experience.

Among the quality criteria included in the structural model are the coefficient of determination (R^2^) and the prediction variance (Q^2^) [56]. The coefficient of determination indicates the proportion of the variance of an endogenous construct explained by its preceding constructs. R^2^ can assume values between 0 and 1 and has a value limit of 0.100 [85]. The predictive power of the model for the ATB, IN, and BV constructs is generally considered good to very good (see Table 2). When testing the R^2^-values of the endogenous latent variable, the R^2^ values for intention (0.422) and behavior (0.510) were considered moderate, while and the R^2^-value of ATB (0.237) was considered weak [58,86]. The model thus explained 23.7% of the variance of ATB, 42.2% of the variance of IN, and 51% of the variance of BV. The coefficient of determination for ATB, IN and BV was clearly above the limit of 0.100 in all three cases, so the effects of the constructs on each other could be classified as significant.

The Q^2^ values determined by blindfolding provided information on how precisely the path model can predict data that were not contained in the model [56]. The model had predictive significance if the Q^2^ values determined were higher than 0 [87]. BV (0.290) had the highest prediction accuracy, followed by IN (0.278) and ATB (0.203). The forecast reliability of the endogenous latent variables could, therefore, also be classified as good. However, as with any theory, weaknesses must be discussed. For example, Jonas and Doll [88] claimed that the TPB is too simple and should be extended using related constructs. We added two constructs (DE and IE). Furthermore, Verplanken and Aarts [89] highlighted the limited use of the TPB in cases of habitual behavior. This fact did not necessarily apply to the construction process under discussion.

Our survey was based on the assumptions that any pig housing with higher FAW standards would be approved and that the operating results would remain constant or even increase compared to previous ones. As many pig farmers, especially those in the regions of North Rhine-Westphalia and Lower Saxony, are affected by not being granted a permit for the construction or conversion of pig housing [25], the survey indicated that this prerequisite for a permit was given. Thus, the actual behavior did not have to be excluded from the model because the construct “behavior” describes the actual construction of a barn to improve animal welfare. By mainly excluding economic factors from this survey, the TPB model was shown to be an appropriate conceptual framework for the study conducted. Economic viability was excluded because many studies showed that performance expectations or financial concerns and risks are dominant influencing factors on farmers’ decisions [15,90,91]. In addition, another study showed that farmers’ concerns related to the financial returns of costly and highly specific investments were unfounded, as farmers participating in animal welfare programs rated their own financial situations as equivalent to those of their colleagues not participating in animal welfare programs [92]. Thus, more research is needed in this other area, assuming that the aforementioned influencing factors are satisfied. In other words, our study considered the framework conditions that actually make pig housing construction financially interesting. As we took for granted the economic viability and constructional approval of new pig housing, the results showed that the construct of “attitude” towards the construction of pig housing also influenced the construction decision. Walder et al. [93] found that self-direction and hedonistic values (cf. our attitude and subjective norm), in contrast with economic- and achievement-based values, are associated with more innovative capabilities. Since indirect experience also has a significant influence on intention, it would be possible to provide more practical information (and inspiration) from other pig farmers on pig housing with higher FAW. It is recommended that relevant networks such as the research project “virtual stall of the future” [94] be established or used further. Farmers could then be convinced by positive examples or share and exchange their experiences and hardships. It is important to specifically promote and advertise model farms and test farms while transforming pig farming. However, transparency must be improved, as economic pressure on farms is growing rapidly. Our study also showed that social norms have a strong effect on the intention to construct pig housing with higher FAW. Social identity could be particularly important in situations where one’s the behavior is “visible” and would thus contribute to a pig farmer’s image. This insight could be used to guide the direction of future efforts to encourage the widespread implementation of pig housing construction. This shows that farmers not only act as individuals on their own farms but are also integrated into their social networks. This means that politics and the promotion of FAW pig housing should offer “presentable” and “easily marketable” options. It is possible that social norms are shaped by the farmers’ desire for recognition of their work, which most farmers currently feel is lacking. The promotion of FAW is not only related to animal welfare but to a greater appreciation of farmers’ work. This has to be achieved within society, and FAW pig housing is a good place to start. As discussed above, the influence of PBC was only minor. TPB is used to explain both reversible and future behavior [69,94] and previously performed behavior [95]. Almost 60% of the variance in behavior can be explained by the determinants included in this model.

However, it is also possible that not all pig farmers who indicate a willingness to construct pig housing with higher FAW standards would actually construct one (behavioral gap) [96]. In addition, by conducting our survey online, some groups of farmers may had been reached more easily than others. On the one hand, online surveys can produce a representativeness problem. However, representativeness can be improved by using different recruitment methods [97]. This was the case for the survey used in this study, as an attempt was made to reach the respondents via several online channels (see Section 3.1. Sampling and Study Design). On the other hand, there are differences in people’s use of the internet. Women, older people, and less educated people use the internet less frequently than men, younger people, and more educated people [98]. This survey may seem to focus too much on North Rhine-Westphalia and Lower Saxony regions. However, the majority of pig farmers in Germany also live in these areas. Futher, significantly more full-time farmers took part in the survey (92%) than part-time farmers (8%). Since there was a clear deviation here from the national average (more part-time farmers), large-scale firms were also characterized as being more willing to innovate [99]. Other results could possibly be obtained by including more part-time farmers. It is also unclear which of the surveyed pig farmers are truly interested in animal welfare. The attitude towards animal welfare could have distorted the sample, if, for example, only a few of the farmers actually supported animal welfare. Moreover, the farmers in our survey had a high level of education (87.2% held degrees or had a PhD). In our study, however, the level of education did not influence construction behavior. Walder et al. [93], analyzed agricultural education as a key driver in farmers’ innovative capacity. This innovative capacity could be equated with the step of constructing pig housing to improve FAW. The majority of those who responded were likely already interested in the topic, were more educated, were open to new ideas, and were looking for alternatives. At the same time, the well-educated pig farmers who responded may also have rejected change processes in housing systems because of the uncertainties mentioned. As education in modern agriculture in Germany will continue to increase rather than decrease in the future, this target group will likely be the most influential in the future. Therefore, our results are important now to establish a baseline.

## 5. Conclusions

Pig housing with increased FAW standards can sustainably improve animal health and behavior while also contributing to socially acceptable livestock farming. However, from a pig farmer point of view, the benefits associated with the construction of animal welfare pig housing are often overshadowed by the many challenges involved. At present, there is little research on the factors, other than economic aspects, that influence the decision to construct such pig housing. Many farmers want to develop their pig housing further but do not know how [3]. Our study, therefore, provides the first detailed insight into the psychological factors that influence pig farmers to carry out the construction of pig housing with higher FAW standards. By applying an extended version of the TPB, we were able to identify attitude, subjective norm, perceived behavioral control, and direct and indirect experience as the main determinants of the intention to construct pig housing to improve FAW. It made sense to extend the model with variables related to past behavior (direct and indirect experience). The behavior of farmers could thus be explained under one model like TPB with additional constructs. However, for each new situation, an assessment must be made to determine which constructs can and cannot be applied.

Therefore, if one excludes licensing problems and the risk of a deteriorating operating result due to the construction of pig housing for the improvement of animal welfare, it becomes clear that other factors also have a significant influence on pig farmers’ behaviors. This produces influencing factors (attitude, subjective norm and experience) that are an important influence on compelling farmers to implement pig housing with higher FAW standards. While our study focused on the psychological factors underlying pig farmers’ behaviors, which can also be described as pig farmers’ behavioral willingness, further research could divide the pig farmers into groups and assess the behavioral intention of each group. It would then be possible to distinguish between pig farmers who have already converted from those who have not converted and thus investigate whether there are differences between the groups in the influences on their intentions and behaviors [38]. A distinction could be made between groups of pig farmers with more (or less) awareness or between pig farmers with weak or strong intentions [94,100,101]. It may also be possible to identify a link between farmers’ levels of education or positions on the farm and their construction behavior. We could then determine, which target group of farm managers needs to be addressed and motivated for future conversion efforts. It is very likely that the implementation of an appropriate draft law to change building regulations and financial incentives would trigger initial changes in behavior related to the construction of pig barns. However, these measures on their own are not enough to yield lasting change in pig farmer behavior. If long-term behavioral changes are desired, it is also necessary to gain an understanding of the factors other than financial incentives or licensing requirements that influence pig farmers’ decision-making. In particular, the psychological factors underlying farmers’ behaviors were already shown to be relevant and significant (such as in various agri-environmental measures and animal welfare programs), as the introduction of such measures was not solely dependent on financial motives [15,102,103]. These insights are important for farmers and upstream industry and politics. Based on these results, a positive attitude alongside positive experiences can facilitate changes by exchanging ideas with colleagues who have already made similar positive changes. Stall construction companies also have a stake in this rethinking process. In this context, the marketing of pig housing must change farmers’ ways of thinking, positive experiences in the area must be shared, and pig farmers’ attitudes and subjective norms must be considered. Moreover, the relevant politics are at a turning point and need more concrete direction. It is not sufficient to make legal decisions on pig farming; farmers must be involved transparently and on a psychological level.

## Figures and Tables

**Table 1:** Cut-off levels of the quality criteria of the reflective measurement models [56]. AVE = average variance extracted.

**Internal Consistency Reliability**	Cronbach’s alpha between 0.6 and 0.9 [56]
	Composite Reliability (CR) between 0.7 and 0.95 CR > Cronbach’s alpha [56]
**Convergent Validity**	Indicator reliability (loadings) > 0.708 [56]
	AVE > 0.5 [56]
**Discriminatory Validity**	Heterotrait–monotrait ratio (HTMT) of the correlations < 0.90 [62] 95% confidence interval ≠ 1 Fornell–Larcker criterion > AVE
	Cross loadings < loadings on the associated construct

Source: Authors’ illustration.

**Table 2:** Evaluation of the inner model (*n* = 424).

Construct	R^2^	Q^2^
ATB	0.237	0.203
IN	0.422	0.278
BV	0.510	0.290

ATB = attitude towards behavior; IN = intention; BV = behavior; R^2^ = coefficient of determination; Q^2^ = prediction variance. Note: Cut-off level: R^2^ > 0.100; Q^2^ > 0. Source: Authors’ calculations and illustration.

## Appendix A

**Table A1:** Profiles of the respondents, *n* = 424.

Variable and Description	Frequency	Percentage (%)
**Gender in%**		
Female	52	87.8
Male	373	12.2
**Age in%**		
18 to 24	25	5.9
25 to 34	92	21.6
35 to 44	87	20.5
45 to 54	116	27.3
55 to 64	95	22.4
65 and older	10	2.4
**Forms of occupation**		
Full time farmer	391	92
Part time Farmer	34	8
**German state**		
North Rhine-Westphalia	171	40.2
Lower Saxony	92	21.6
Bavaria	40	9.4
Hesse	26	6.1
Schleswig–Holstein	26	6.1
other	70	16.5

**Table A2:** Descriptive results for the applied indicators and outer model evaluation.

Construct	Corresponding Item	Outer Loading	Mean (SD)
**Attitude Towards Behavior**			
	ATB1: By building pig housing to improve FAW, I feel personally better.	0.940 ***	3.101 (1.33)
	ATB2: Constructing pig housing to improve FAW on my farm gives me a better conscience towards my animals.	0.933 ***	2.842 (1.37)
**Subjective Norm**			
	SN1: Professional colleagues are largely admired for constructing pig housing to improve FAW.	0.835 ***	3.219 (1.04)
	SN2: Most people who mean something to me believe that I should construct pig housing to improve FAW.	0.831 ***	2.675 (1.23)
	SN3: If I were to construct pig housing to improve FAW, most people I care about would admire this step.	0.896 ***	3.285 (1.20)
**Perceived Behavioral Control**			
	PBC1: I do not have enough time to construct pig housing to improve FAW.	0.738 ***	2.365 (1.17)
	PBC2: Other pig farmers don’t construct pig housing to improve FAW, so I don’t have to.	0.775 ***	1.659 (0.91)
	PBC3: For me, a pig housing construction projects to improve FAW are too complicated at the moment.	0.745 ***	2.904 (1.37)
**Intention**			
	IN1: In the near future, I would like to consider constructing pig housing to improve FAW. Attention: If you have constructed or reconstructed within the last 5 years, please click on “agree completely”.	0.802 ***	3.515 (1.44)
	IN2: I would like to start constructing pig housing to improve FAW soon. Attention: If you have constructed or reconstructed within the last 5 years, please click on “agree completely”.	0.879 ***	3.209 (1.57)
	IN3: (Also) in the future I intend to operate pig housing to improve FAW on my farm.	0.815 ***	3.675 (1.20)
**Behavior**			
	BV1: I have already constructed pig housing to improve FAW on my farm recently.	0.765 ***	2.508 (1.64)
	BV2: I have already applied for a permit for the construction of pig housing to improve FAW on my farm.	0.786 ***	1.856 (1.48)
	BV3: I am currently constructing pig housing to improve FAW.	0.761 ***	2.216 (1.53)
**Direct Experience**			
	DE1: I was involved in at least one construction project for pig housing to improve FAW.	0.908 ***	2.864 (1.72)
	DE2: I have had positive experiences with the construction of pig housing to improve FAW.	0.925 ***	2.835 (1.40)
**Indirect Experience**			
	IE1: I have or have had contact with people who have already implemented the construction of pig housing to improve FAW.	0.893 ***	3.640 (1.37)
	IE2: I have or have had contact with people who have had positive experiences with constructing pig housing to improve FAW.	0.936 ***	3.289 (1.30)

Outer loadings > 0.7; asterisks indicate different levels of significance (*** *p* < 0.01); the mean: descriptive analysis with a five-point Likert scale from 5 (totally agree) to 1 (totally disagree); *n* = 424.

**Table A3:** Internal consistency: Cronbach’s alpha, composite reliability, and average variance extracted from the constructs.

Construct	Cronbach’s Alpha	Composite Reliability (CR)	Average Variance Extracted (AVE)
Attitude	0.860	0.934	0.877
Subjective norm	0.815	0.890	0.730
Perceived behavioral control	0.618	0.795	0.564
Intention	0.780	0.872	0.694
Behavior	0.662	0.815	0.594
Direct experience	0.809	0.913	0.840
Indirect experience	0.808	0.911	0.837

Cutoff levels: Cronbach’s alpha > 0.7 (0.6); composite reliability (CR) = 0.6–0.9; average variance Extracted (AVE) > 0.5; *n* = 424.

**Table A4:** Discriminant validity: heterotrait–monotrait ratio (HTMT).

Construct	DE	ATB	IE	IN	SN	BV	PBC
DE							
ATB	0.578						
IE	0.450	0.286					
IN	0.641	0.634	0.465				
SN	0.453	0.822	0.412	0.613			
BV	0.764	0.474	0.480	0.903	0.412		
PBC	0.405	0.345	0.297	0.425	0.394	0.366	

HTMT of the correlations < 0.90; ATB = attitude towards behavior; BV = behavior; DE = direct experience; IN = intention; IE = indirect experience; PBC = perceived behavioral control; SN = subjective norm.

**Table A5:** Results of the structural model and hypothesis testing, *n* = 424.

H_0_		Path Coefficients	T-Statistic ^a^	Support H_0_
ATB → IN	H1	0.221 ***	3.611	Supported
SN → IN	H2	0.169 ***	2.997	Supported
PBC → IN	H3	−0.081 **	2.021	Supported
PBC → BV	H4	0.016	0.484	Not supported
IN → BV	H5	0.486 ***	12.932	Supported
DE → ATB	H6	0.487 ***	12.199	Supported
DE → IN	H7	0.274 ***	5.403	Supported
DE → BV	H8	0.299 ***	6.876	Supported
IE → IN	H9	0.143 ***	3.043	Supported
IE → BV	H10	0.061 *	1.733	Supported

ATB = attitude towards behavior, BV = behavior, DE = direct experience, IE = indirect experience, IN = intention, SN = subjective norm, PBC = perceived behavior control; ^a^ calculated by bootstrapping (5000 subsamples); asterisks indicate different levels of significance (*** *p* < 0.01, ** *p* < 0.05, * *p* < 0.10).
